# Recent advances of epilepsy associated with neurofibromatosis type 1

**DOI:** 10.3389/fneur.2025.1640309

**Published:** 2025-08-15

**Authors:** Ying Ren, Wandong Hu, Song Su, Qi Zhang, Wenchao Zhang, Hongwei Zhang, Guangyu Wang

**Affiliations:** ^1^Department of Neurology, Children’s Hospital Affiliated to Shandong University, Jinan, China; ^2^Department of Neurology, Jinan Children’s Hospital, Jinan, China; ^3^Department of Neurosurgery, Children’s Hospital Affiliated to Shandong University, Jinan, China; ^4^Department of Neurosurgery, Jinan Children’s Hospital, Jinan, China

**Keywords:** neurofibromatosis type 1, NF1, epilepsy, neurofibromin, Ras/Raf/MAPK pathway

## Abstract

**Background and aim:**

Neurofibromatosis type 1 (NF1) is an autosomal dominant tumor predisposition syndrome caused by pathogenic variants in the NF1 gene. It exhibits highly variable and unpredictable clinical manifestations involving multiple organ systems, with café-au-lait macules and multiple neurofibromas being hallmark features. Epilepsy represents a common central nervous system complication in NF1, though its underlying mechanisms remain poorly understood. NF1 patients with epilepsy exhibit a higher prevalence of developmental delay and learning disabilities. Early identification and personalized therapy are critical for optimal management of this patient population. This review aims to synthesize published literature on the disease, thereby providing a comprehensive, detailed, and updated overview of its entire clinical spectrum.

**Methods:**

We conducted a comprehensive literature search in PubMed, China National Knowledge Infrastructure, and Chinese Medical Association Journal Full-text Database for original research articles with available full-text manuscripts in English or Chinese, with a publication cutoff date of March 1, 2025. Our search strategy employed the terms “neurofibromatosis type 1” OR “NF1” combined using the Boolean operator AND with “epilepsy” OR “seizure.” Priority was given to studies published in the last decade, though seminal earlier research was also incorporated.

**Results:**

An extensive bibliography was researched and summarized in the review. Epilepsy represents a common central nervous system complication in NF1, though its underlying mechanisms remain poorly understood. NF1-associated epilepsy demonstrates diverse seizure semiologies, with focal seizures being the most prevalent phenotype. Although the majority of patients demonstrate favorable responses to oral anti-seizure medications, those with structural brain abnormalities frequently develop drug-resistant epilepsy. Notably, a subset of these patients may achieve significant seizure reduction or complete remission through surgical intervention when the epileptogenic zone is clearly delineated. Furthermore, while targeted therapies remain an active area of investigation, their application in NF1-associated epilepsy remains supported only by case report-level evidence.

**Conclusion:**

This review comprehensively summarizes current knowledge regarding the pathogenesis, clinical characteristics, diagnostic approaches, and therapeutic strategies for NF1-related epilepsy, aiming to optimize diagnostic accuracy and treatment outcomes for affected individuals.

## 1 Introduction

Neurofibromatosis type 1 (NF1) is an autosomal dominant tumor predisposition syndrome caused by pathogenic variants in the NF1 gene, with an estimated prevalence of 1/4,000 to 1/2,000 individuals without significant racial or gender predilection. This multisystem disorder typically manifests in early childhood, exhibiting highly variable and unpredictable clinical features. Hallmark characteristics include café-au-lait macules and multiple neurofibromas. In addition, it can be accompanied by a variety of benign and malignant tumors, skeletal dysplasia, cardiovascular and cerebrovascular diseases, cognitive and psychological disorders, epilepsy (1, 2). The prevalence of epilepsy in NF1 ranges from 4–14% (3), significantly higher than the general population (1–2%) (4). Seizure semiology is diverse, encompassing focal motor seizures, absence seizures, generalized tonic–clonic seizures, epileptic spasms and so on, with focal seizures being most prevalent (5, 6). NF1-related epilepsy is usually secondary to intracranial lesions, such as tumors, hippocampal sclerosis (HS), or focal cortical dysplasia (FCD) (7, 8), while showing no established association with undefined bright objects (UBOs) or optic pathway gliomas (5–7). Notably, structural lesions cannot account for all cases, as over 50% of NF1-related epilepsy patients lack identifiable structural abnormalities (5, 8), suggesting that the genetic condition itself may predispose to neuronal hyperexcitability and epileptogenesis (6). Despite growing interest in rare diseases and recent advancements in NF1 research, most reports on NF1-associated epilepsy remain limited to case studies. Therefore, this review systematically examines the pathogenesis, clinical characteristics, diagnostic approaches, and therapeutic strategies for NF1-related epilepsy, aiming to enhance clinical management and improve patient outcomes.

## 2 Methods

We conducted a comprehensive literature search in PubMed, China National Knowledge Infrastructure, and Chinese Medical Association Journal Full-text Database for original research articles with available full-text manuscripts in English or Chinese, with a publication cutoff date of March 1, 2025. Our search strategy employed the terms “neurofibromatosis type 1” OR “NF1” combined using the Boolean operator AND with “epilepsy” OR “seizure.” Priority was given to studies published in the last decade, though seminal earlier research was also incorporated.

## 3 Pathogenesis of NF1-related epilepsy

The NF1 gene, located at 17q11.2, contains 58 exons and encodes neurofibromatosis protein, which is a GTP-activating protein expressed in many cell types including neurons, astrocytes, and oligodendrocytes. The most important feature of NF1 protein is that it is a Ras-GTP activating protein, which can activate the Ras/Raf/MAPK pathway (4). The Ras/Raf/MAPK pathway is one of the most well-characterized pathways in cell biology that mediates transmembrane receptor signaling, critically involved in modulating cell proliferation, apoptosis, and essential gene expression. The specific reason for the increased incidence of epilepsy in NF1 patients remains unclear. But, first of all, structural abnormalities of the brain are one of the main causes of epilepsy in NF1 patients. NF1 patients with epilepsy are more likely to have brain structural abnormalities than normal people, such as dysembryoplastic neuroepithelial tumors (DNET), HS, FCD, low-grade glioma, and neuronal heterotopia. Studies have proved that somatic variation during cortical development has become an important cause of epilepsy (9). NF1 patients are born with an inactivated NF1 allele, and tumors develop when the second allele becomes somatically inactivated due to loss of heterozygosity or a second mutational event (10). Khoshkhoo et al. confirmed this theory. They detected NF1 somatic variants that activate the Ras/Raf/MAPK pathway in the hippocampus of two patients with drug-resistant mesial temporal lobe epilepsy (11), both of whom had a germline mutation in the NF1 gene and were diagnosed with NF1. The postoperative pathology of both patients showed mesial temporal lobe sclerosis, and one of them was accompanied by low-grade epilepsy related tumor (11). In addition, activation of MAP kinase signaling pathway has also been found in gangliogliomas, including NF1 biallelic variants (12). This “second-hit” theory has also been demonstrated in animal models. Because homozygous NF1 mutant mice do not survive, traditional NF1 animal models are heterozygous, but the tumor tissues of these heterozygous mice show the loss of wild-type NF1 gene (13). The “second-hit” theory has also been widely recognized in structural epilepsy caused by activation of mTOR signaling pathway (14). Although somatic variants in the “two-hit” theory cannot be detected in all cases, accumulating evidence has demonstrated its role in NF1-associated epilepsy. Moreover, it has been suggested that second-hit environmental events, such as acute immune activation induced by lipopolysaccharide injection early in life, may promote epilepsy in NF1+/ -mice and may be a risk factor for NF1 related epilepsy (4). Therefore, “second hit” may be one of the pathogenesis of NF1-related epilepsy.

**Figure 1:**
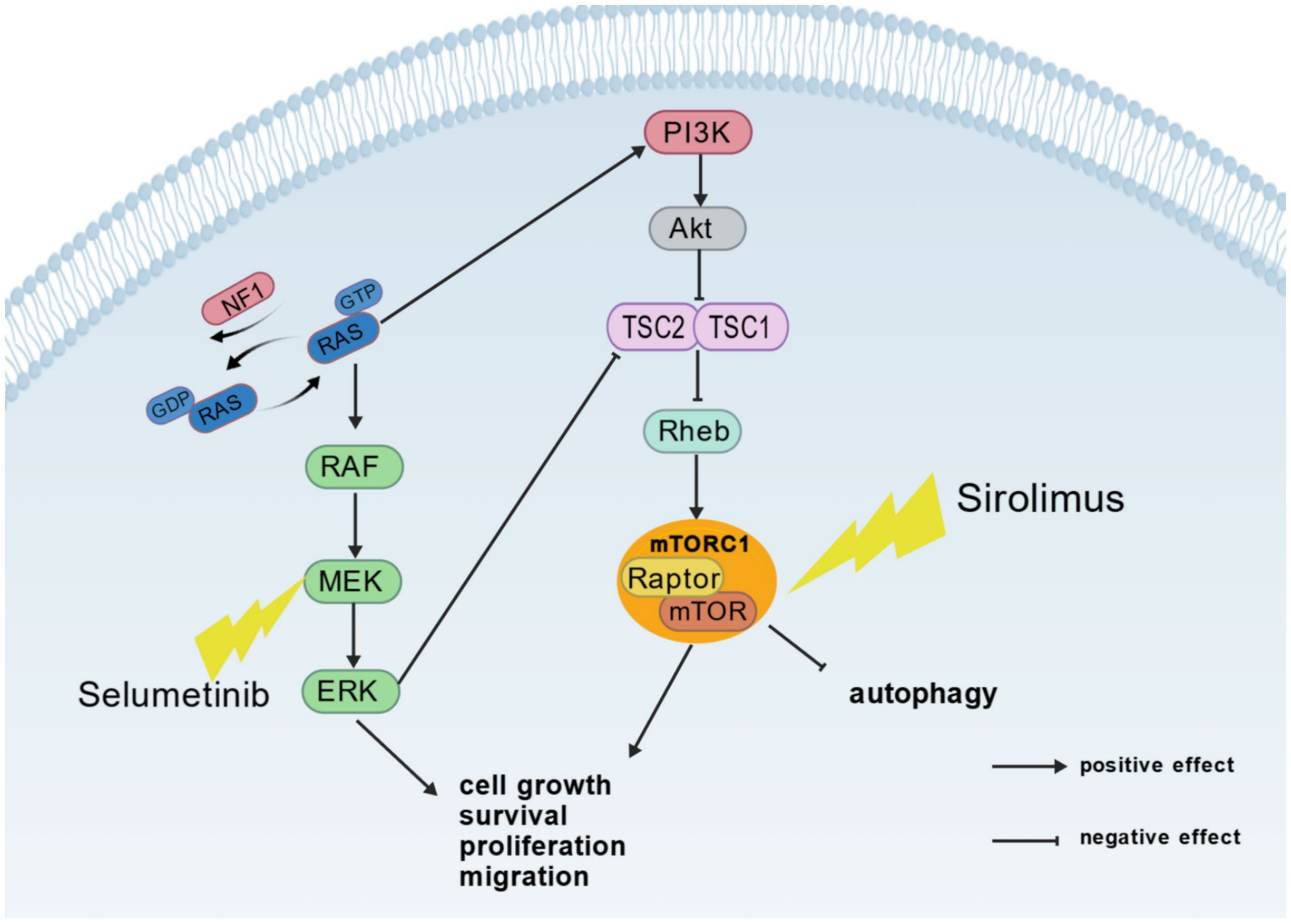
The Ras/Raf/MAPK and mTOR signaling pathways and their crosstalk. RAS, rat sarcoma viral oncogene homolog; GDP, guanosine diphosphate; GTP, guanosine triphosphate; NF1, neurofibromatosis 1; ERK: extracellular signal-regulated kinase; MEK: mitogen activated protein kinase kinase; Raf: rapidly accelerated fibrosarcoma; PI3K, Phosphatidylinositol 3-Kinase; Akt, Protein Kinase B; TSC1/TSC2, Tuberous Sclerosis Complex 1/2; Rheb, Ras Homolog Enriched in Brain; Raptor, Regulatory-Associated Protein of mTOR; mTOR, Mammalian Target of Rapamycin; mTORC1, Mechanistic Target of Rapamycin Complex 1. Created with biogdp.com.

Brain structural abnormalities have been implicated in seizures in NF1 patients, but they do not explain seizures in all cases of NF1-related epilepsy. More than half of NF1-related epilepsy cases do not have clear structural lesions (5). These seizures may result from dysregulation of endogenous electrical brain activity, suggesting that genetic variants themselves may contribute to epilepsy in these patients. An increased incidence of seizures in animal models of NF1 without known macroscopic or neoplastic intracerebral lesions was demonstrated by Sabetghadam et al. (6); thus providing an evidence that genetic variants themselves play a role in epilepsy or seizures in NF1 patients. This conclusion was also supported by other researchers (15). First of all, neurofibroma protein is a multi-domain protein, and its main feature is Ras-GTP activating protein (4). It catalyzes the hydrolysis of GTP-Ras to GDP-Ras and is a negative regulator of the Ras-pathway. The reduction or complete absence of neurofibromin caused by NF1-related pathogenic variants can lead to excessive activation of Ras-signaling, which in turn increases the downstream signal release of the MAPK pathway, ultimately stimulating transcription and cell growth (16–21). Activated Ras-signaling leads to cross-activation of the PI3K-mTOR pathway, another important pathway for cell proliferation and survival (15). On the one hand, GTP-bound Ras can bind and activate PI3K; on the other hand, ERK can also promote mTOR activation by phosphorylating TSC2 (13) (Figure 1). It is well known that tuberous sclerosis, which can increase the incidence of seizures, is associated with hyperactivity of the mTOR pathway (22). Therefore, the over-activation of MAPK pathway and mTOR pathway may be one of the potential causes of seizures in NF1 patients. Secondly, enhanced ERK signaling has been reported in the NF1+/− mouse model, and the enhanced ERK signaling can stimulate NMDA receptor expression (4, 23, 24). If the trafficking of NMDA receptors is increased in NF1, an excitation/inhibition imbalance may occur, resulting in excessive excitation and, in turn, the development of epilepsy. Finally, Ras-signaling in the dendritic spines of pyramidal neurons is required for various forms of synaptic plasticity (25). The Ras-PI3K-mTOR signaling pathway also plays a central role in the regulation of dendritic spine morphology. Moreover, NF1 protein can also participate in the formation of filopodia and dendritic spines through other pathways (26). The over-activation of Ras-signaling caused by NF1 gene variants leads to synaptic plasticity and neuronal activity damage (25, 26), thus participating in the pathogenesis of epilepsy. Other potential mechanisms of NF1-related epilepsy may also be related to changes in ion channels and abnormalities in neurotransmitters (27, 28). Due to alterations in GABAergic signaling and dysfunction of sodium, potassium, calcium and HCN channels, the brains of NF1 mice exhibit a state of hyperexcitability (13). Previous reports have described changes in voltage-gated ion channels associated with NF1. For example, calcium currents are increased in hippocampal neurons of NF1+/ -mice, which may mediate the increased neuronal excitability (4, 15). A distinct study demonstrated a progressive increase in NF1 protein expression in the pilocarpine-induced epileptic rat model. Furthermore, NF1 was found to facilitate the progression of epileptic seizures. The underlying mechanisms remain unclear but may involve NF1-mediated regulation of GABAergic neurotransmission and dendritic spine formation. These findings suggest that dysregulation of neural network activity, whether due to enhanced or diminished NF1 protein function, is closely associated with epileptogenesis (26).

Although more and more studies have demonstrated the important role of NF1 protein in neurodevelopment, the specific mechanism of epilepsy susceptibility in NF1 patients has not been fully elucidated. At present, it appears that there is no evidence for an unique and singular mechanism of epileptogenesis in patients with NF1. Neurotransmitter release, neuronal differentiation, cortical development, regulation of ion channel function, synapse formation and synaptic activity involved in NF1 proteins are all crucial for the occurrence of epilepsy. Further studies are needed to explain the role of NF1 gene and protein in nervous system development and the mechanism of epilepsy.

## 4 Clinical features of NF1-related epilepsy

The prevalence of refractory epilepsy in patients with NF1 is less common than in the setting of other neurocutaneous disorders such as tuberous sclerosis (29). Compared with NF1 patients without epilepsy, NF1 patients with epilepsy are more likely to have brain structural abnormalities on MRI, and the temporal lobe is the most susceptible brain region. In a study of 12 surgically treated NF1 patients with drug-resistant epilepsy, 10 cases (83.3%) demonstrated temporal lobe onset, with 8 patients (66.7%) achieving seizure-free state at 1-year follow-up (17). The most common postoperative pathology was DNET (5 cases) and HS (4 cases) (17). Gangliogliomas, neuronal heterotopia, FCD, and cerebrovascular lesions have been reported in NF1 patients with epilepsy (5, 29), especially in patients with refractory epilepsy. Many NF1 patients show focal lesions with increased signal intensity on T2-weighted MRI, which has been named UBO by foreign scholars. UBO can occur in nearly 90% of NF1 patients, mostly in children under 15 years of age, and most commonly involved sites are basal ganglia, thalamus, brain stem, or cerebellum (8, 30, 31). However, whether UBO can increase the risk of epilepsy in NF1 patients has not been confirmed, because previous studies have shown conflicting results (32), and currently it is more inclined to believe that UBO is not associated with epilepsy (33). Overall, not all NF1 brain lesions have been definitively identified as epileptic foci. Previous reports of NF1-related epilepsy have highlighted the lack of agreement between structural lesions visible on MRI and epileptogenic zones (34). Many reports of NF1-related epilepsy lack a correlation between electroencephalogram (EEG), clinical presentation, and brain imaging findings to confirm the epileptogenicity of structural lesions. These findings underscore the necessity for comprehensive data collection and multidisciplinary discussions in the management of all refractory epilepsy cases, including NF1-associated epilepsy. Personalized treatment strategies are crucial for symptom control and quality-of-life improvement.

It has been reported that nearly half of NF1 patients with epilepsy have no structural abnormalities on MRI (5, 6). Moreover, MRI lesions in patients are not always co-localized with epileptic discharges on EEG (6, 35). The seizure forms of NF1-related epilepsy are mainly focal seizures, and generalized seizures and epileptic encephalopathy have also been reported (32). There are relatively few studies on the EEG discharge patterns in patients with NF1, abnormal EEG may be registered in approximately 25% of NF1 patients (29, 36), and focal epileptiform activity during interictal period of seizures are the most common discharge patterns (37). Generalized discharges, hypsarrhythmia have been occasionally reported in NF1 patients (32). This is consistent with the type of seizure presented. However, if the EEG is re-examined in a short period of time, it may be found that the EEG abnormalities are dynamic and may evolve into different epileptic discharge patterns. Patel et al. once detected the evolution process from focal epileptic discharge to generalized epileptic discharge and then to hypsarrhythmia on the EEG of a child with NF1 and epilepsy (38). More clinical data and basic experiments are needed to confirm the formation and spreading of interictal discharge patterns in NF1 patients.

NF1 is an autosomal dominant disease, and about 50% of the children with NF1 inherit a pathogenic gene variant from their parents (13). Some studies have pointed out that the incidence of epilepsy will increase if the gene mutation is inherited from the mother (39), and some studies have overturned this conclusion. The specific correlation still needs to be verified by a large sample in the future. In addition, some studies have shown that NF1 patients with epilepsy have a higher incidence of truncating variants and a lower incidence of missense variants (33). This may be because the former leads to the destruction of the structure of the gene coding product and is more likely to cause more severe clinical phenotype including epilepsy. NF1 patients with epilepsy exhibit a higher prevalence of developmental delay and learning disabilities (8, 35, 40). It also suggests that NF1 patients with epilepsy may be more in need of clinical help. The selection of antiepileptic drugs for NF1 patients with epilepsy is not uniform. Clinically, it is still recommended to choose reasonable antiepileptic drugs according to seizure forms and epilepsy syndromes. Several studies support (5, 8) that NF1-related epilepsy has a good prognosis, with most children achieving seizure-freedom after treatment with one or two antiepileptic drugs. It is not easy to control seizures in NF1 patients with epilepsy caused by intracranial tumors or HS and other structural lesions, but epilepsy surgery can be effectively used for NF1 patients with refractory epilepsy if a clear epileptogenic zone is identified (17).

## 5 Diagnostic points of NF-related epilepsy

The National Institutes of Health(NIH) has established a set of clear diagnostic criteria for NF1 in 1987 (1), but a study involving 1,893 NF1 patients under the age of 21 showed that 46% of patients with sporadic NF1 under the age of 1 year did not meet the diagnostic criteria established by the NIH (2). However, almost all children with NF1 meet these criteria by the age of 8 years (41). Therefore, in 2021, the International Consensus Group on Neurofibromatosis Diagnostic Criteria proposed to revise the NF1 diagnostic criteria established in 1987 (1, 42). The updated diagnostic criteria for NF1 state that at least two of the following criteria must be present to confirm the diagnosis (1): (1) Six or more café-au-lait macules over 5 mm in greatest diameter in prepubertal individuals and over 15 mm in greatest diameter in postpubertal individuals. (2) Freckling in the axillary or inguinal region. (3) Two or more neurofibromas of any type or one plexiform neurofibroma. (4) Optic pathway glioma. (5) Two or more iris Lisch nodules identified by slit lamp examination or two or more choroidal abnormalities (CAs)-defined as bright, patchy nodules imaged by optical coherence tomography (OCT) /near-infrared reflectance (NIR) imaging. (6) A distinctive osseous lesion such as sphenoid dysplasia, anterolateral bowing of the tibia, or pseudarthrosis of a long bone. (7) A heterozygous pathogenic NF1 variant with a variant allele fraction of 50% in apparently normal tissue such as white blood cells. For those without parental history of NF1, two or more of the seven clinical features mentioned above can be diagnosed as NF1. Patients with parental history of NF1 can be diagnosed if one or more clinical features are met. The diagnosis of epilepsy in NF1 patients is consistent with the principles and procedures of conventional epilepsy, and it is necessary to combine the patient’s medical history, EEG, brain imaging and other test results. The forms of seizures are classified based on the semiological description of seizures, EEG, imaging, seizure videos, etc. (43).

## 6 Treatment of NF1-related epilepsy

### 6.1 Antiepileptic drugs

For NF1 patients with epilepsy, seizure control mainly depends on antiepileptic drugs. Many studies (5, 8, 34) have shown that NF1-related epilepsy has a good prognosis, and most children can achieve seizure-free state after treatment with one or two antiepileptic drugs (44). Among the 12 patients with NF1-related epilepsy in Khair and his colleagues’s cohort, 75% had seizure control with an oral antiepileptic drug. Serdaroglu et al. (35) and Santoro et al. (8) reported that 50–65% of NF1-related epilepsy patients obtained seizure control after oral antiepileptic drug. Studies in China also support the above data. Among 13 NF1 patients with epilepsy reported by Wu et al. (33), 8 patients were free from seizures after using 1–2 antiepileptic drugs. However, there is no definite guidelines for the selection of antiepileptic drugs in NF1-related epilepsy, and the appropriate antiepileptic drugs are mainly selected according to the type of seizures or epilepsy syndrome.

### 6.2 Surgical treatment

In NF1 patients with epilepsy could be due to structural lesions such as intracranial tumors or HS, seizure control may be challenging. However, epilepsy surgery can be effectively employed in such cases if a well-defined epileptogenic zone is identified. Barba et al. (17) reported a series of 12 NF1 patients with drug-resistant epilepsy, among whom 11 exhibited structural brain abnormalities, including HS and tumors. Postoperatively, 8 patients achieved seizure freedom state at the 1-year follow-up. Similarly, Pecoraro et al. identified 26 epilepsy cases among 184 NF1 patients, noting that all 3 patients with medial temporal lobe sclerosis who underwent temporal lobectomy experienced seizure free state (7). Therefore, for NF1 patients with refractory epilepsy—particularly those with structural lesions such as HS or intracranial tumors—epilepsy surgery represents a viable therapeutic option, provided a thorough preoperative evaluation confirms a well-localized epileptogenic focus.

### 6.3 Ketogenic diet

The ketogenic diet (KD) is a specialized dietary regimen characterized by low carbohydrate intake, high fat content, and moderate to low protein consumption (tailored to individual needs), effectively promoting ketogenesis. As an established therapeutic intervention, KD demonstrates remarkable efficacy in the management of drug-resistant epilepsy across all age groups (45, 46). In response to the considerable attrition rates observed with traditional KD protocols, researchers have developed several modified formulations designed to mitigate adverse effects while optimizing treatment adherence and patient acceptability. Currently, four principal ketogenic dietary therapies are recognized in clinical practice: the classic ketogenic diet (cKD), the modified Atkins diet (MAD), the medium chain triglyceride ketogenic diet (MCTKD), and low glycemic index treatment (LGIT). While these variants differ substantially in their macronutrient composition, current evidence does not support significant differences in therapeutic efficacy among them (47). Importantly, less restrictive protocols appear to confer advantages in terms of long-term compliance without compromising therapeutic outcomes. Studies indicate that the overall responder rate for KD ranges from 13 to 70%, or even higher (45). Notably, emerging evidence suggests that KD may provide additional benefits beyond seizure control, including measurable improvements in cognitive function and behavioral outcomes (48, 49).

Although multiple potential mechanisms of the KD have been elucidated, the fundamental basis for its anticonvulsant effects remains incompletely understood. The primary therapeutic advantage of this intervention lies in its metabolic shift from carbohydrate to fat utilization as the main energy source. This profound alteration in energy metabolism induces widespread adaptations across multiple biochemical pathways. Notably, inhibition of the mTOR signaling pathway has emerged as one plausible mechanism underlying the KD’s efficacy in refractory epilepsy. Animal studies have substantiated this mechanism (50, 51), where the expression of pS6 and pAkt, markers of mTOR pathway activation, was reduced in hippocampus and liver of rats fed KD. Furthermore, clinical observations have reported particularly favorable outcomes when employing the KD for refractory epilepsy cases associated with mTOR pathway dysregulation (46).

Current evidence indicates that aberrant mTOR pathway activation plays a significant role in NF1-associated epilepsy, particularly in refractory cases. This mechanistic understanding suggests that the KD may represent a promising therapeutic intervention for this patient population. However, while the KD has been widely adopted in clinical practice for various forms of epilepsy, robust clinical data specifically supporting its efficacy and safety in NF1-related epilepsy remain lacking. Further well-designed clinical trials and mechanistic studies are warranted to establish its therapeutic potential in this specific indication.

### 6.4 Targeted therapy

It is well established that the NF1 protein catalyzes the hydrolysis of GTP-Ras to GDP-Ras, serving as a negative regulator of the Ras-pathway. Pathogenic variants in the NF1 gene can lead to excessive activation of Ras-signaling, subsequently increasing downstream signaling of the MAPK pathway, namely the Ras–Raf–Mek–Erk cascade (52). Selumetinib is a potent, selective, orally administered, ATP-noncompetitive inhibitor of MEK1 and MEK2. It binds to MEK1/2 proteins, inducing conformational changes that disrupt cellular signal transduction, thereby inhibiting tumor cell growth and proliferation (52). Since its approval, selumetinib has been widely used in inoperable NF1 patients with plexiform neurofibromas, demonstrating significant therapeutic efficacy (53). However, selumetinib has not been approved for the treatment of NF1-related epilepsy. Cantor et al. (54) reported a case of a patient with NF1, drug-resistant epilepsy, and extensive optic pathway glioma who achieved dose-dependent seizure control while on selumetinib. Notably, seizure recurrence was observed after dose reduction due to adverse effects, but seizure control was restored upon dose re-escalation. Similarly, Sarah Barrière observed complete seizure cessation and significant improvement in interictal electroencephalographic abnormalities in patients treated with MEK inhibitors for optic pathway gliomas (21). Nevertheless, current evidence supporting selumetinib for NF1-related epilepsy remains limited to case reports, necessitating further clinical studies to validate its efficacy. Another promising therapeutic approach involves inhibitors of the PI3K-AKT–mTOR signaling pathway. As previously mentioned, hyperactivated Ras-signaling can lead to cross-activation of the PI3K-mTOR cascade (15). Preclinical studies have demonstrated that AKT or PI3K inhibitors suppress the progression of astrocyte and optic gliomas in NF1-deficient mice (55). Clinically, the mTOR inhibitor sirolimus has been used in NF1-associated tumors, such as delaying plexiform neurofibroma progression or alleviating tumor-related pain (56). Other targeted agents, including the tyrosine kinase inhibitor imatinib, have also been explored for NF1-related tumors (57). Despite the growing use of targeted therapies for NF1-associated tumors, there is currently insufficient evidence supporting their efficacy in NF1-related epilepsy. Further research is needed to evaluate their potential role in seizure management for this patient population.

## 7 Conclusion

NF1 is a multisystem genetic disorder associated with a significantly higher prevalence of epilepsy compared to the general population. NF1-related epilepsy demonstrates substantial clinical heterogeneity and complex pathogenic mechanisms. Nevertheless, the overall therapeutic outcomes are favorable, with the majority of patients achieving seizure control through anti-seizure medications. Patients with structural abnormalities exhibit an increased propensity to develop drug-resistant epilepsy. However, comprehensive presurgical evaluation to identify well-defined epileptogenic zones may enable effective surgical intervention with significant seizure improvement. The molecular mechanisms underlying epileptogenesis in pediatric NF1 patients remain incompletely understood. Current targeted therapy research has primarily focused on NF1-associated tumors. Strengthening translational research bridging basic science and clinical practice is crucial for improving long-term outcomes and quality of life in NF1 patients with epilepsy. Future investigations should further elucidate the pathogenesis and contributing factors of NF1-related epilepsy to develop more precise therapeutic strategies for this patient population.
